# Long-term clinical course and outcomes of patients with microscopic polyangiitis-associated interstitial lung disease

**DOI:** 10.3389/fphar.2023.1064307

**Published:** 2023-01-30

**Authors:** Min Jee Kim, Donghee Lee, Jooae Choe, Jin Woo Song

**Affiliations:** ^1^ Department of Internal Medicine, Asan Medical Center, University of Ulsan College of Medicine, Seoul, South Korea; ^2^ University of Ulsan College of Medicine, Seoul, South Korea; ^3^ Department of Radiology, Asan Medical Center, University of Ulsan College of Medicine, Seoul, South Korea; ^4^ Department of Pulmonary and Critical Care Medicine, Asan Medical Center, University of Ulsan College of Medicine, Seoul, South Korea

**Keywords:** interstitial lung disease, microscopic polyangiitis, survival, prognosis, risk factor

## Abstract

**Background:** Interstitial lung disease (ILD) is a significant complication associated with microscopic polyangiitis (MPA) that has a poor prognosis. However, the long-term clinical course, outcomes, and prognostic factors of MPA-ILD are not well defined. Hence, this study aimed to investigate the long-term clinical course, outcomes, and prognostic factors in patients with MPA-ILD.

**Methods:** Clinical data of 39 patients with MPA-ILD (biopsy proven cases, *n* = 6) were retrospectively analyzed. High resolution computed tomography (HRCT) patterns were assessed based on the 2018 idiopathic pulmonary fibrosis diagnostic criteria. Acute exacerbation (AE) was defined as the worsening of dyspnea within 30 days, with new bilateral lung infiltration that is not fully explained by heart failure or fluid overload and that does not have identified extra-parenchymal causes (pneumothorax, pleural effusion, or pulmonary embolism).

**Results:** The median follow-up period was 72.0 months (interquartile range: 44–117 months). The mean age of the patients was 62.7 years and 59.0% were male. Usual interstitial pneumonia (UIP) and probable usual interstitial pneumonia patterns on high resolution computed tomography were identified in 61.5 and 17.9% of the patients, respectively. During the follow-up, 51.3% of patients died, and the 5- and 10-year overall survival rates were 73.5% and 42.0%, respectively. Acute exacerbation occurred in 17.9% of the patients. The non-survivors had higher neutrophil counts in bronchoalveolar lavage (BAL) fluid and more frequent acute exacerbation than the survivors. In the multivariable Cox analysis, older age (hazard ratio [HR], 1.07; 95% confidence interval [CI], 1.01–1.14; *p* = 0.028) and higher BAL counts (HR, 1.09; 95% CI, 1.01–1.17; *p* = 0.015) were found to be the independent prognostic factors associated with mortality in patients with MPA-ILD.

**Conclusion:** During the 6 years-follow-up, about half of patients with MPA-ILD died and approximately one-fifth experienced acute exacerbation. Our results suggest that older age and higher BAL neutrophil counts mean poor prognosis in patients with MPA-ILD.

## 1 Introduction

Microscopic polyangiitis (MPA) is a non-granulomatous necrotizing vasculitis involving small vessels and a type of antineutrophil cytoplasmic antibody-associated vasculitis (AAV) (Chung and Seo, 2010). Lung and renal involvement are the most common complications in patients with MPA and have a significant impact on survival (Corral-Gudino et al., 2011). While the kidney is the most commonly affected organ, lung involvement can be observed in 25%–55% of patients with MPA (Chung and Seo, 2010). Although interstitial lung disease (ILD) is a rare manifestation in patients with MPA, it is associated with poor survival (Fernandez Casares et al., 2015).

In previous studies, the long-term survival of patients with MPA-ILD was reported to range from 50% to 60% at 5 years; additionally, the main cause of death was worsening of pulmonary fibrosis (PF), which accounts for 40%–60% of cases (Homma et al., 2004; Hervier et al., 2009; Comarmond et al., 2014; Fernandez Casares et al., 2015). The presence of myeloperoxidase (MPO) specific antineutrophil cytoplasmic antibodies (ANCA), a usual interstitial pneumonia (UIP) pattern on high resolution computed tomography (HRCT), and induction therapy with glucocorticoids have been identified as unfavorable prognostic factors in patients with MPA-ILD (Homma et al., 2013; Comarmond et al., 2014; Fernandez Casares et al., 2015; Watanabe et al., 2019). However, the long-term clinical course and the prognostic factors of MPA-ILD are still not well defined. Therefore, this study aimed to investigate the clinical course, outcomes, and prognostic factors in patients with MPA-ILD.

## 2 Materials and methods

### 2.1 Study population

Between January 2000 and December 2019, 65 patients with MPA were diagnosed at Asan Medical Center, Seoul, South Korea. ILD was confirmed on HRCT images in 39 patients (biopsy proven cases, *n* = 6) and they were included in this study. All patients were diagnosed with MPA based on the European Medicines Agency algorithm and Chapel Hill Consensus Conference criteria (Watts et al., 2007; Jennette et al., 2013). The study was approved by the Institutional Review Board of Asan Medical Center (2019-0861), and the requirement for informed consent was waived due to the retrospective nature of this study.

### 2.2 Clinical data

The clinical and survival data were retrospectively collected from medical records and/or the records of the National Health Insurance of Korea. The spirometric parameters, total lung capacity (TLC) by plethysmography, and diffusing capacity of the lung for carbon monoxide (DL_CO_) were measured according to the American Thoracic Society (ATS)/European Respiratory Society (ERS) recommendations (Macintyre et al., 2005; Miller et al., 2005; Wanger et al., 2005). The results were expressed as percentages of normal predicted values. A 6-min walk test (6MWT) was performed according to the ERS/ATS recommendations (Holland et al., 2014). Bronchoalveolar lavage (BAL) was performed according to the ATS guidelines (Meyer et al., 2012).

The data from follow-up visits at 3–6-month intervals or from hospitalization were reviewed to determine the development of complications, such as acute exacerbation (AE), pneumonia, diffuse alveolar hemorrhage (DAH), or malignant tumors. AE was defined based on the criteria proposed by Collard et al. (2016), as a worsening of dyspnea within 30 days, with new bilateral lung infiltration that is not fully explained by heart failure or fluid overload and that does not have any identified extra-parenchymal causes (pneumothorax, pleural effusion, or pulmonary embolism). Pneumonia was defined as focal or unilateral lung infiltration with identified causative organism; however, when a causative pathogen was not identified and infection was strongly suspected clinically (symptoms such as purulent sputum, rapid and significant response to antibiotic treatment alone), it was also diagnosed as pneumonia (Kwon et al., 2022). DAH was defined according to the following criteria: 1) diffuse ground-glass opacity and/or consolidation on HRCT without alternative cause 2) hemoptysis, bronchoscopic evidence of hemorrhage, or bloody BAL fluid (Hozumi et al., 2021). Disease progression or improvement was defined as a relative decline or increase of at least 10% of the forced vital capacity (FVC) predicted value (% predicted) (Richeldi et al., 2012). The changes in lung function that did not meet these criteria were classified as stabilization. The relative FVC changes were calculated as follows: (FVC % predicted at follow-up − FVC % predicted at baseline)/FVC % predicted at baseline × 100 (%).

### 2.3 HRCT image evaluation

HRCT scans were performed in accordance with standard protocols at full inspiration without contrast enhancement. The HRCT scanned images were reviewed by a radiologist (J.C.) without the clinical and pathologic information provided. Overall, HRCT patterns were categorized as UIP, probable UIP, indeterminate UIP, or alternative diagnosis, based on the idiopathic pulmonary fibrosis (IPF) diagnostic criteria (Raghu et al., 2018). Based on the HRCT findings, the patients were also classified based on whether they had a UIP-like pattern or not. A UIP-like pattern was diagnosed when HRCT findings were consistent with a UIP or probable UIP pattern (Jacob et al., 2019). A UIP-like pattern was defined as a reticular pattern with traction bronchiectasis or bronchiolectasis with or without honeycombing and the absence of findings compatible with a UIP pattern, such as extensive ground-glass opacities, micro-nodules, discrete cysts, or segmental/lobar consolidations.

### 2.4 Statistical analysis

All data are expressed as mean ± standard deviation (SD) or median [interquartile range (IQR)] for continuous variables and as percentages for categorical variables. The continuous variables were compared using the Student’s t-test or Mann-Whitney *U* test, and categorical variables were compared using Pearson’s chi-squared or Fisher’s exact test. A Kaplan-Meier survival analysis and the log-rank test were performed to analyze the survival rate. The survival time was calculated as the number of months from the date of ILD diagnosis until death or the end of the follow-up period. The patients were censored if they were alive on 21 July 2022. The Cox analysis was performed to identify the prognostic factors for mortality in patients with MPA-ILD. In the multivariable Cox analysis, baseline variables with *p*-value < 0.1 in the unadjusted analysis were included in the multivariable models. All *p*-values were two-tailed, with the statistical significance set at a *p*-value < 0.05. All statistical analyses were performed using IBM SPSS Statistics for Windows, Version 26.0 (IBM Corp., Armonk, NY, United States).

## 3 Results

### 3.1 Baseline characteristics

**FIGURE 1:**
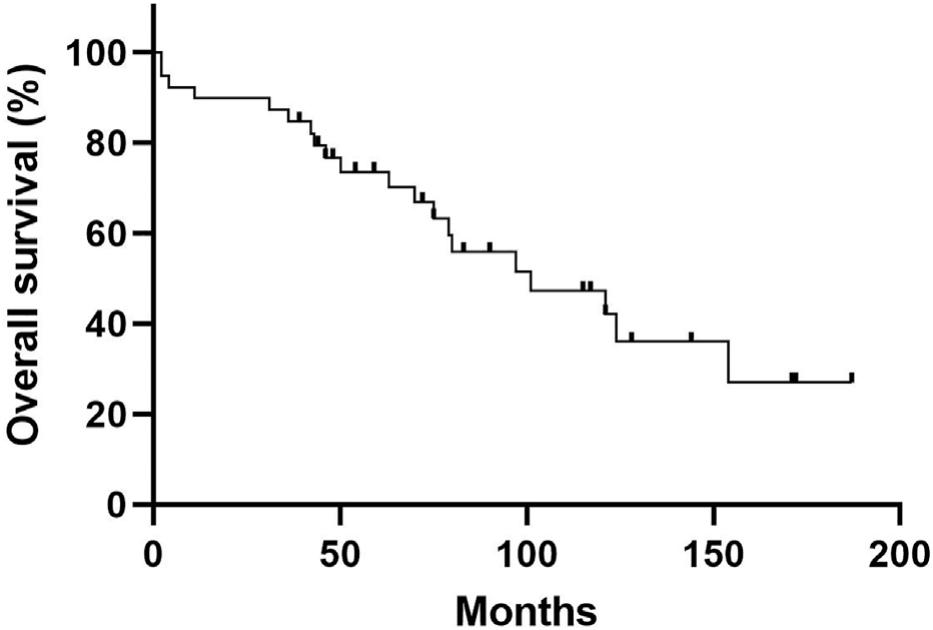
Overall Kaplan—Meier survival curve of patients with MPA-ILD. The 5-year and 10-year overall survival rates of patients with MPA-ILD were 73.5% and 42.0%, respectively. MPA, microscopic polyangiitis; ILD, interstitial lung disease.

The baseline characteristics of the study population are shown in Table 1. The mean age was 62.7 years, and 59.0% of patients were male. The median follow-up period was 72.0 months (IQR: 44.0–117.0 months), and 20 patients (51.3%) died. The 1-, 5-, and 10-year cumulative survival rates of patients were 89.7, 73.5, and 42.0%, respectively (Figure 1). Overall, ILD was the initial manifestation of MPA in 66.7% of patients, and extra-pulmonary organ involvement was found in 92.3% of patients, of which renal involvement was the most common, accounting for 83% of extra-pulmonary organ involvement, followed by peripheral nerve involvement (36%). The non-survivors showed older age and higher BAL neutrophil counts than the survivors (Table 1); however, other variables, including lung function and exercise capacity, did not vary between the two groups.

**TABLE 1:** Comparison of baseline characteristics between the non-survivors and survivors among patients with MPA-ILD.

	Total	Non-survivors	Survivors	*p-*value
Number of patients	39	20	19	
Age at ILD diagnosis	62.7 ± 9.8	66.0 ± 7.9	59.3 ± 10.6	0.031
Male sex	23 (59.0)	14 (70.0)	9 (47.4)	0.151
Ever-smokers	21 (53.8)	12 (60.0)	9 (47.4)	0.429
Sequence of diagnosis				
ILD first	26 (66.7)	15 (75.0)	11 (57.9)	0.257
MPA first	1 (2.6)	1 (5.0)	0	>0.999
Concurrence	12 (30.8)	4 (20.0)	8 (42.1)	0.135
Extra-pulmonary involvement				
Kidney	30 (76.9)	15 (75.0)	15 (78.9)	>0.999
Peripheral nervous system	13 (33.3)	8 (40.0)	5 (26.3)	0.365
Autoantibody				
MPO-ANCA titer, IU/mL	116.0 (38.0–134.0)	121.0 (64.0–154.0)	116.0 (32.0–134.0)	0.221
MPO-ANCA positivity	36 (92.3)	18 (90.0)	18 (94.7)	>0.999
ANA positivity	5 (12.8)	2 (10.5)	3 (16.7)	0.660
CRP, mg/dL	6.9 ± 6.7	7.8 ± 6.9	5.9 ± 6.6	0.386
Pulmonary function test				
FVC, % predicted	77.0 ± 15.3	75.8 ± 15	78.3 ± 16	0.625
DLco, % predicted	59.8 ± 14.8	60.6 ± 17.4	58.9 ± 11.9	0.733
TLC, % predicted	78.4 ± 13.1	78.7 ± 14.6	78.1 ± 11.6	0.887
6-min walk test				
Distance, meter	392.8 ± 143.8	360.3 ± 154.0	427.0 ± 127.6	0.162
The lowest SpO_2_, %	93.0 ± 4.9	92.2 ± 5.8	93.8 ± 3.7	0.331
Bronchoalveolar lavage				
WBC/mm^3^	317.9 ± 300.9	295.1 ± 254	345.6 ± 358.0	0.650
Neutrophil, %	10.9 ± 8.7	13.1 ± 9.8	7.6 ± 6.0	0.110
Lymphocyte, %	15.0 ± 9.0	15.7 ± 10.7	14.2 ± 6.7	0.639

The data are presented as mean ± standard deviation, median (interquartile range), or number (%).

MPA, microscopic polyangiitis; ILD, interstitial lung disease; MPO-ANCA, myeloperoxidase-antineutrophil cytoplasmic antibodies; ANA, antinuclear antibody; CRP, C-reactive protein; FVC, forced vital capacity; DLco, diffusing capacity of the lung for carbon monoxide; TLC, total lung capacity; WBC, white blood cell; SpO_2_, oxygen saturation measured by pulse oximetry.

### 3.2 Clinical course

Table 2 presents the clinical course of patients with MPA-ILD. Pneumonia was the most frequent pulmonary complication (23.1%), followed by AE, DAH, and lung cancer. AE occurred in 7 (17.9%) patients during the follow-up, and all of them died. During the follow-up, the non-survivors developed AE more frequently than the survivors (Table 2). However, there was no difference in the development of pneumonia, DAH, and lung cancer between the two groups.

**TABLE 2:** Comparison of the clinical course between the non-survivors and survivors among patients with MPA-ILD.

	Total	Non-survivors	Survivors	*p*-value
Number of patients	39	20	19	
Complications				
Pneumonia	9 (23.1)	6 (30.0)	3 (15.8)	0.501
Diffuse alveolar hemorrhage	7 (17.9)	3 (15.0)	4 (21.1)	0.695
Acute exacerbation	7 (17.9)	7 (35.0)	0	0.015
Lung cancer	3 (7.7)	3 (15.0)	0	0.231
FVC changes (6 months), % predicted	2.9 ± 13.7	1.4 ± 14.1	4.4 ± 13.6	0.541
FVC changes (12 months), % predicted	2.7 ± 13.2	−0.9 ± 13.5	6.4 ± 12.1	0.117
Pulmonary function change				
Improved	7 (21.2)	4 (23.5)	3 (18.8)	>0.999
Stabilization	20 (60.6)	8 (47.1)	12 (75.0)	0.101
Progression	6 (18.2)	5 (29.4)	1 (6.3)	0.175

The data are presented as mean ± standard deviation or number (%).

MPA, microscopic polyangiitis; ILD, interstitial lung disease; FVC, forced vital capacity.

Overall, pulmonary function was stabilized in 60.6% of patients, improved in 21.2% of patients, and progressed in 18.2% of patients (Table 2). The changes in lung function did not vary between the non-survivors and survivors; however, the non-survivors showed numerically lower improvement in FVC during the 6- or 12-month follow-up periods than the survivors without statistical significance.

In terms of treatment, 38 (97.4%) patients received steroid alone (*n* = 12) or with cyclophosphamide (*n* = 26) as an induction therapy. Maintenance therapy included steroid only (*n* = 8) or immunosuppressant (*n* = 30; azathioprine = 22, mycophenolate mofetil = 5, methotrexate = 2, cyclosporine = 1) with or without steroid.

### 3.3 Clinical course according to the HRCT patterns

Of all patients, 24 (61.5%) were classified as having a UIP pattern on HRCT, 7 (17.9%) as having probable UIP, 5 (12.8%) as having indeterminate UIP, and 3 (7.7%) as having an alternative diagnosis. A UIP-like pattern on HRCT was identified in 31 (79.5%) patients. The baseline characteristics of patients according to the HRCT patterns are summarized in Table 3. Patients with a UIP-like pattern showed a tendency for higher BAL neutrophil counts than those without; however, there was no difference in the frequency of complications between the two groups except a tendency for a higher prevalence of lung cancer in patients with a non-UIP-like pattern (Table 4). There was no significant difference in the baseline lung function between the two groups; however, patients with a UIP-like pattern showed numerically lower improvement in FVC over a 6- or 12-month follow-up period without statistical significance (Tables 3, 4).

**TABLE 3:** Comparison of the baseline characteristics according to the HRCT patterns in patients with MPA-ILD.

	Total	UIP-like	Non-UIP-like	*p*-value
Number of patients	39	31	8	
Age at ILD diagnosis	62.7 ± 9.8	63.6 ± 9.2	59.4 ± 11.8	0.281
Male sex	23 (59.0)	19 (61.3)	4 (50.0)	0.694
Ever-smokers	21 (53.8)	18 (58.1)	3 (37.5)	0.432
Sequence of diagnosis				
ILD first	26 (66.7)	20 (64.5)	6 (75.0)	0.694
MPA first	1 (2.6)	1 (3.2)	0	>0.999
Concurrence	12 (30.8)	10 (32.3)	2 (25.0)	>0.999
Extra-pulmonary involvement				
Kidney	30 (76.9)	23 (74.2)	7 (87.5)	0.653
Peripheral nervous system	13 (33.3)	10 (32.3)	3 (37.5)	>0.999
Autoantibody				
MPO-ANCA titer, IU/mL	116.0 (38.0–134.0)	108.0 (35.0–134.0)	134.0 (87.5–165.0)	0.094
MPO-ANCA positivity	36 (92.3)	29 (93.5)	7 (87.5)	>0.999
ANA positivity	5 (12.8)	3 (10.3)	2 (25.0)	0.564
CRP, mg/dL	6.9 ± 6.7	7.4 ± 7.1	4.8 ± 4.6	0.358
Pulmonary function test				
FVC, % predicted	77.0 ± 15.3	79.0 ± 14.7	69.4 ± 16.1	0.116
DLco, % predicted	59.8 ± 14.8	58.6 ± 14.3	69.4 ± 17.0	0.317
TLC, % predicted	78.4 ± 13.1	80.1 ± 13.1	71.3 ± 11.3	0.110
6-min walk test				
Distance, meter	392.8 ± 143.8	382.0 ± 153.6	439.0 ± 83.5	0.352
The lowest SpO_2_, %	93.0 ± 4.9	93.1 ± 4.8	92.6 ± 5.4	0.813
Bronchoalveolar lavage				
WBC/mm^3^	317.9 ± 300.9	348.0 ± 326.9	189.0 ± 78.0	0.249
Neutrophil, %	10.9 ± 8.7	11.9 ± 8.7	7.5 ± 8.8	0.290
Lymphocyte, %	15.0 ± 9.0	13.5 ± 7.2 1	21.3 ± 13.3	0.216

Data are presented as mean ± standard deviation, median (interquartile range) or number (%).

MPA, microscopic polyangiitis; ILD, interstitial lung disease; MPO-ANCA, myeloperoxidase-antineutrophil cytoplasmic antibodies; ANA, antinuclear antibody; CRP, C-reactive protein; FVC, forced vital capacity; TLC, total lung capacity; DLco, diffusing capacity of the lung for carbon monoxide; WBC, white blood cell; SpO_2_, oxygen saturation measured by pulse oximetry.

**TABLE 4:** Comparison of the clinical course according to the HRCT patterns in patients with MPA-ILD.

	Total	UIP-like	Non-UIP-like	*p*-value
Number of patients	39	31	8	
Complications				
Pneumonia	9 (23.1)	6 (19.4)	3 (37.5)	0.538
Diffuse alveolar hemorrhage	7 (17.9)	6 (19.4)	1 (12.5)	>0.999
Acute exacerbation	7 (17.9)	6 (19.4)	1 (12.5)	>0.999
Lung cancer	3 (7.7)	1 (3.2)	2 (25.0)	0.101
FVC changes (6 months), % predicted	2.9 ± 13.7	1.1 ± 10.6	11.1 ± 22.8	0.338
FVC changes (12 months), % predicted	2.7 ± 13.2	2.2 ± 12.3	4.9 ± 17.8	0.662
Pulmonary function change				
Improvement	7 (17.9)	5 (18.5)	2 (33.3)	0.584
Stabilization	20 (51.3)	17 (63.0)	3 (50.0)	0.659
Progression	6 (15.4)	5 (18.5)	1 (16.7)	>0.999

Data are presented as mean ± standard deviation or number (%).

MPA, microscopic polyangiitis; ILD, interstitial lung disease; FVC, forced vital capacity.

**FIGURE 2:**
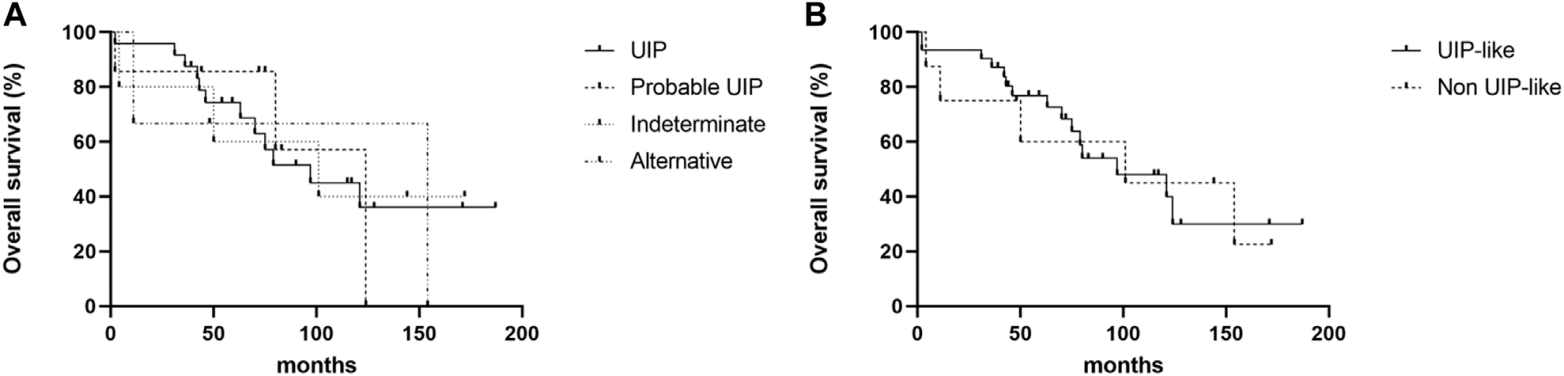
Kaplan—Meier survival curve of patients with MPA-ILD according to HRCT patterns. **(A)** Comparison of overall survival according to the HRCT patterns, **(B)** Comparison of overall survival between patients with UIP-like pattern and non-UIP-like pattern. **(A)** There were no differences in overall survival rates according to the HRCT patterns; the median survival period was 97.0 months for a UIP pattern, 124.0 months for probable UIP, 101.0 months for indeterminate UIP, and 154.0 months for alternative diagnosis (log-rank test, *p* = 0.985). **(B)** There was no difference in the overall survival between patients with UIP-like and non-UIP-like pattern on HRCT; the median survival period was 97.0 months for UIP-like pattern, and 101.0 months for non-UIP-like pattern (log-rank test, *p* = 0.969). MPA, microscopic polyangiitis; ILD, interstitial lung disease; HRCT, high resolution computed tomography; UIP, usual interstitial pneumonia.

The Kaplan—Meier survival curve of patients with MPA-ILD according to the HRCT patterns is shown in Figure 2. There was no statistical difference in overall survival according to the HRCT patterns in patients with MPA-ILD (Figure 2A); the median survival period was 97.0 months for a UIP pattern, 124.0 months for probable UIP, 101.0 months for indeterminate UIP, and 154.0 months for alternative diagnosis (*p* = 0.985). There was also no difference in the overall survival between patients with UIP-like and non-UIP-like patterns on HRCT (median survival period, 97.0 vs. 101.0 months, *p* = 0.969) (Figure 2B).

### 3.4 Risk factors for mortality

In the unadjusted Cox analysis, older age, male sex, higher BAL neutrophil counts, AE, and greater changes in the FVC (12 months) were significant prognostic factors for mortality in patients with MPA-ILD (Table 5). In the multivariable Cox analysis, older age (hazard ratio [HR], 1.072; 95% confidence interval [CI], 1.008–1.141; *p* = 0.028) and higher BAL neutrophil counts (HR, 1.091; 95% CI, 1.017–1.170; *p* = 0.015) were independent prognostic factors in patients with MPA-ILD.

**TABLE 5:** Prognostic factors for mortality in patients with MPA-ILD assessed by Cox regression analysis.

	Unadjusted analysis		
Variables	HR	95% CI	*p*-value
Age at ILD diagnosis	1.081	1.018–1.148	0.011
Male sex	2.294	0.871–6.039	0.093
MPA first	3.072	0.392–24.075	0.285
Ever smoker	1.767	0.714–4.374	0.218
CRP mg/dL	1.030	0.969–1.095	0.342
FVC, % predicted	0.993	0.968–1.018	0.575
DLco, % predicted	1.009	0.979–1.040	0.561
TLC, % predicted	1.001	0.967–1.036	0.950
6MWT, lowest SpO_2_, %	0.936	0.860–1.018	0.121
6MWT, distance (meter)	0.999	0.996–1.003	0.743
BAL, Neutrophil, %	1.075	1.006–1.149	0.034
BAL, Lymphocyte, %	0.993	0.940–1.049	0.795
UIP-like pattern	0.980	0.349–2.755	0.969
FVC changes (6 months), % predicted	0.977	0.939–1.016	0.243
FVC changes (12 months), % predicted	0.952	0.907–0.999	0.045
Acute exacerbation	4.203	1.616–10.933	0.003
Multivariate analysis^a^			

Variables	HR	95% CI	*p*-value
Age at ILD diagnosis	1.072	1.008–1.141	0.028
Male sex	2.190	0.699–6.866	0.179
BAL, Neutrophil, %	1.091	1.017–1.170	0.015

ILD: interstitial lung disease, MPA: microscopic polyangiitis, CRP: C-reactive protein, FVC: forced vital capacity, DLco: diffusing capacity of the lung for carbon monoxide, TLC: total lung capacity, 6MWT: 6-min walk test, SpO_2_: oxygen saturation measured by pulse oximetry, BAL: bronchoalveolar lavage, UIP: usual interstitial pneumonia.

^a^ Only baseline variables with *p*-value < 0.1 in the unadjusted analysis were entered into the multivariable models.

## 4 Discussion

In this study, during a follow-up of 6 years, about half of patients with MPA-ILD died and approximately one-fifth of patients experienced AE. Our results suggest that older age and higher BAL neutrophil counts were independent prognostic factors for mortality in patients with MPA-ILD. However, HRCT patterns were not associated with prognosis in patients with MPA-ILD.

In our study, the clinical characteristics of patients with MPA-ILD were similar to those in previous reports (Tzelepis et al., 2010; Huang et al., 2014; Fernandez Casares et al., 2015). In a previous study including nine patients with MPA and PF (MPA-PF) (Fernandez Casares et al., 2015), there was a slight predominance of males (55.6%), and our study also revealed a male predominance of 59.0%. In our study, 92.3% of patients presented MPO-ANCA positivity, and the development of ILD preceded that of vasculitis in 66.7% of patients. These features corresponded with those reported in the study by Fernandez Casares et al. (2015); they showed that 32.1% of 28 patients with MPA had ILD, and ILD was the initial manifestation in 55.5% of those with ILD. Likewise, ILD preceded extra-pulmonary manifestations in other studies (Tzelepis et al., 2010; Huang et al., 2014). Tzelepis et al. (2010) compared the clinical characteristics and outcomes of patients with MPA with and without ILD, and revealed that ILD preceded extra-pulmonary manifestations in 53.8% of patients with MPA-ILD (*n* = 13). Additionally, Huang et al. (2014) reported that ILD was observed to precede the diagnosis of MPA in 68.4% of 19 patients with MPA-ILD.

In our study, higher BAL neutrophil counts were associated with poor prognosis in patients with MPA-ILD. The significant difference in neutrophil count between the non-survivors and the survivors might be explained by the correlation of increase in BAL neutrophils with disease severity and prognosis of IPF (Meyer et al., 2012). A previous study by Foucher et al. (1999) also suggested that the MPO antibody-related autoimmune response contributes to lung injury after the local release of products of activated neutrophils, which supports a pathogenic role of MPO antibody in pulmonary lesions. Moreover, a study by Birnbaum et al. (2007) involving patients with MPA-PF, showed that vasculitis with neutrophilic degranulation may have triggered fibroblastic foci and the development of ILD. Consistently, a recent study by Koike et al. (2022) comparing sural nerve biopsy specimens between patients with MPA and those with non-systemic vasculitic neuropathy (NSVN) demonstrated that neutrophils adhere to vascular endothelial cells, migrate to the extravascular spaces, and release neutrophil components in the extracellular spaces in patients with MPA; however, these findings were not observed in those with NSVN.

Pulmonary complications, including DAH, chronic respiratory failure, and AE, are significant causes of death and are associated with a high mortality rate in patients with MPA-ILD (Hozumi et al., 2021). PF with respiratory failure contributes to 40%–100% of deaths in patients with MPA-ILD (Tzelepis et al., 2010; Comarmond et al., 2014; Huang et al., 2014; Fernandez Casares et al., 2015), suggesting that survival is more closely associated with PF than with vasculitis itself. In our study, AE occurred in approximately 20% of patients during follow-up, and all the patients died. The exact incidence of AE in patients with MPA-ILD is not known; however, a previous study, involving 80 patients with MPA-ILD, suggested that the 1-year cumulative incidence of AE was 7.2%, similar to that of IPF (8.5% of 1-year incidence) (Kim et al., 2006; Hozumi et al., 2021). In the unadjusted Cox regression analyses of our study, AE was a significant risk factor for mortality, which was in line with the results of previous studies demonstrating that AE accounts for up to 20%–60% of the causes of death in patients with AAV-ILD (Hosoda et al., 2016; Watanabe et al., 2019).

In our study, a UIP pattern on HRCT was the dominant HRCT pattern, consistent with previous studies (Arulkumaran et al., 2011; Fernandez Casares et al., 2015). The most common fibrosis pattern on HRCT in patients with MPA-ILD was a UIP pattern, accounting for 77.7% and 57.1% of patients with MPA-ILD in previous studies by Arulkumaran et al. (2011) and Fernandez Casares et al. (2015), respectively. However, unlike other connective tissue disease-associated ILDs (Koo et al., 2019), whether HRCT patterns affect the survival of patients with MPA-ILD has not been clarified. Comarmond et al. (2014) reported that the UIP pattern and atypical UIP or NSIP patterns on HRCT were not associated with an increased risk of mortality in 49 patients with PF associated with AAV (HR, 5.01; 95% CI, 0.63–39.92; *p* = 0.13 and HR, 3.54; 95% CI, 0.21–58.61; *p* = 0.38). Furthermore, in our study, there was no significant difference in the overall survival according to the HRCT patterns in patients with MPA-ILD. Moreover, Hozumi et al. (2021) showed that there was no difference in the overall survival between the UIP group and the non-UIP group in 84 patients with MPA-ILD (5-year cumulative survival rate, 35.8% vs. 54.2%, *p* = 0.18).

Our study has some limitations. First, it was a retrospective observational study performed at a single center, and this may limit the generalizability of our results. However, the baseline characteristics of the subjects were similar to those reported in other studies (Homma et al., 2004; Foulon et al., 2008; Comarmond et al., 2014; Fernandez Casares et al., 2015). Second, the number of patients was small, and this would have limited the ability to produce statistically significant results. The subjects in our study also received various treatments, which may lead to the different patients outcome. However, even considering a small number of patients, our results identify prognostic factors for survival in patients with MPA-ILD. The results of our study need to be confirmed in other studies with larger cohorts. Finally, the definition of a UIP-like pattern used in this study was modified from the HRCT classification of IPF diagnostic guidelines. In the absence of a consensus on the definition of the UIP-like pattern in fibrosing ILDs other than IPF, the modification may be inevitable in some subtypes of ILDs. Despite these limitations, the inclusion of patients with MPA-ILD—rather than just those with ANCA positivity and the assessment of their long-term clinical outcomes are the strengths of this study.

In conclusion, during a follow-up of 6 years, it was observed that about 50% and 20% of patients with MPA-ILD experienced death and AE, respectively. Our results suggest that older age and higher BAL neutrophil counts signify poor prognosis in patients with MPA-ILD.

## Data Availability

The original contributions presented in the study are included in the article/supplementary material, further inquiries can be directed to the corresponding author.

## Abbreviations

AE, Acute exacerbation; ANCA, Antineutrophil cytoplasmic antibodies; ATS, American Thoracic Society; BAL, Bronchoalveolar lavage; DAH, Diffuse alveolar hemorrhage; ERS, European Respiratory Society; HR, Hazard ratio; HRCT, High resolution computed tomography; ILD, Interstitial lung disease; IPF, Idiopathic pulmonary fibrosis; PF, Pulmonary fibrosis; SD, Standard deviation; TLC, Total lung capacity; UIP, Usual interstitial pneumonia.
